# Limited transferability of European-based body mass index and blood pressure polygenic scores to admixed Brazilian cohorts

**DOI:** 10.3389/fmed.2026.1771205

**Published:** 2026-03-13

**Authors:** Ana Luisa Calixto Rodrigues, Samantha Kuwada Teixeira, Antonio Luiz Pinho Ribeiro, Ester Cerdeira Sabino, Isabela Judith Martins Benseñor, Paulo Andrade Lotufo, Jose Eduardo Krieger, Alexandre da Costa Pereira

**Affiliations:** 1 Laboratorio de Genetica e Cardiologia Molecular, Instituto do Coracao (InCor), Hospital das Clinicas (HCFMUSP), Faculdade de Medicina, Universidade de Sao Paulo, Sao Paulo, Brazil; 2 Felício Rocho Hospital, Belo Horizonte, Brazil; 3 Department of Internal Medicine, Telehealth Center, Federal University of Minas Gerais Medical School, Belo Horizonte, Brazil; 4 Department of Infectious Diseases, University of São Paulo Medical School, São Paulo, Brazil; 5 Center for Clinical and Epidemiologic Research, University of São Paulo, São Paulo, Brazil; 6 Department of Medicine, Brigham and Women’s Hospital, Harvard Medical School, Boston, MA, United States

**Keywords:** admixed populations, blood pressure, body mass index, hypertension, obesity, polygenic risk score

## Abstract

**Background:**

Most polygenic risk scores (PRS) are derived and validated using genetic data from European populations. However, European-based PRS perform poorly in individuals of non-European ancestry, and their transferability to the admixed Brazilian population remains unknown.

**Methods:**

PRS derived from the United Kingdom Biobank (UKB) were selected from the PGS Catalog, including 33 scores for body mass index (BMI), 36 for systolic blood pressure (SBP), and 33 for diastolic blood pressure (DBP). PRS were applied to 4,758 participants from two geographically distinct Brazilian cohorts (São Paulo and North Minas Gerais) and a UKB sample. Performance was evaluated across self-identified racial subgroups and in Brazilian individuals genetically similar to the UK sample, as determined by genomic clustering techniques (Uniform Manifold Approximation and Projection, UMAP; and Principal Component Analysis, PCA). Effect sizes were compared using multivariable mixed-effects models.

**Results:**

Most BMI PRS were validated in São Paulo (96.7%) and North Minas Gerais (90.9%), whereas blood pressure PRS showed lower validation rates (SBP: 66.7 and 38.9%; DBP: 69.7 and 54.5%, respectively). Validated PRS consistently exhibited lower effect sizes in Brazilian cohorts compared to the UKB (*p* < 0.001). PRS calibration for obesity and hypertension using cohort-specific quintiles and precision–recall F1 scores did not improve performance. BMI PRS effects were slightly higher in São Paulo than in North Minas Gerais (+0.39 kg/m^2^ per SD, *p* < 0.001), whereas SBP and DBP effects did not differ significantly between regions. BMI PRS effects were higher in Brazilian Whites than Non-Whites (+0.60 kg/m^2^ per SD, *p* < 0.001), but blood pressure PRS effects were similar in the racial subgroups. Across all traits, PRS effects were higher in the UKB than in Brazilian individuals clustering with the British by UMAP/PCA (*p* < 0.001), and no differences were observed between Brazilian UMAP/PCA subgroups.

**Conclusion:**

European-derived PRS are not directly transferable to the Brazilian population without prior empirical validation. Regional origin, self-identified race, and genetic similarity to the UKB do not reliably identify Brazilian subgroups with differential PRS performance. These findings highlight the urgent need for polygenic scores derived and trained on Brazilian genomic data.

## 1 Introduction

Polygenic risk scores (PRS) estimate genetic susceptibility to common diseases and have potential clinical utility for cardiometabolic traits such as coronary artery disease, atrial fibrillation, and type 2 diabetes (1). A major barrier to PRS widespread adoption is that they usually transfer poorly to target populations whose ancestry differs from the discovery cohorts (2). To date, most PRS rely on European ancestry population genetic data (3). Numerous studies have demonstrated that PRS derived from individuals of European ancestry do not predict as well in individuals of non-European ancestry (2–5). For example, across multiple traits, reductions in PRS accuracy of ∼37, ∼50, and ∼78% have been reported in individuals of South-Asian, East-Asian and African ancestries, respectively, relative to individuals of European ancestry (2). Accuracy has been shown to decay with the target population’s genetic distance to the training population and with increasing proportion of admixture (3–6).

Transferability of European-based PRS to the admixed Brazilian population remains poorly established. The demographic history of Brazil is highly complex, resulting in the world’s largest recently admixed population. Although Indigenous populations inhabited the territory prior to European arrival, the admixture processes that shaped the contemporary Brazilian population began with Portuguese colonization in 1,500 AD. The initial colonization process brought over 5 million Europeans to Brazil, alongside the forced migration of at least 4 million enslaved Africans. The indigenous population underwent a profound reduction and was partly incorporated into the broader society through intermarriage (7). Early admixture was sex-biased, mainly between Portuguese men and Indigenous or African women, explaining why 71% of Y-chromosome lineages are European, while most mitochondrial lineages are African or Indigenous (8). The peak of admixture occurred in the 18th–19th centuries, coinciding with a new large-scale wave of European immigration (Portuguese, Italians, Germans, and Spaniards). The second half of the 19th and first half of the 20th centuries saw a substantial influx of Arab and Japanese immigrants (7, 8). This unique genetic background raises uncertainty about the applicability of European-derived PRS in Brazil.

There is limited evidence of how European-based PRS perform in Brazilian cohorts. A trans-ethnic PRS for body mass index (BMI) has shown adequate performance in Brazilians, although with attenuated effects (9). A systolic blood pressure (SBP) PRS derived from the United Kingdom Biobank (UKB) showed weaker associations in Brazilian cohorts (10). Similarly, European-based breast cancer PRS have been validated in Brazilian cohorts, but with attenuated effects (11, 12). These results indicate that the validity of European-based PRS in Brazil cannot be assumed without empirical evaluation.

**FIGURE 1:**
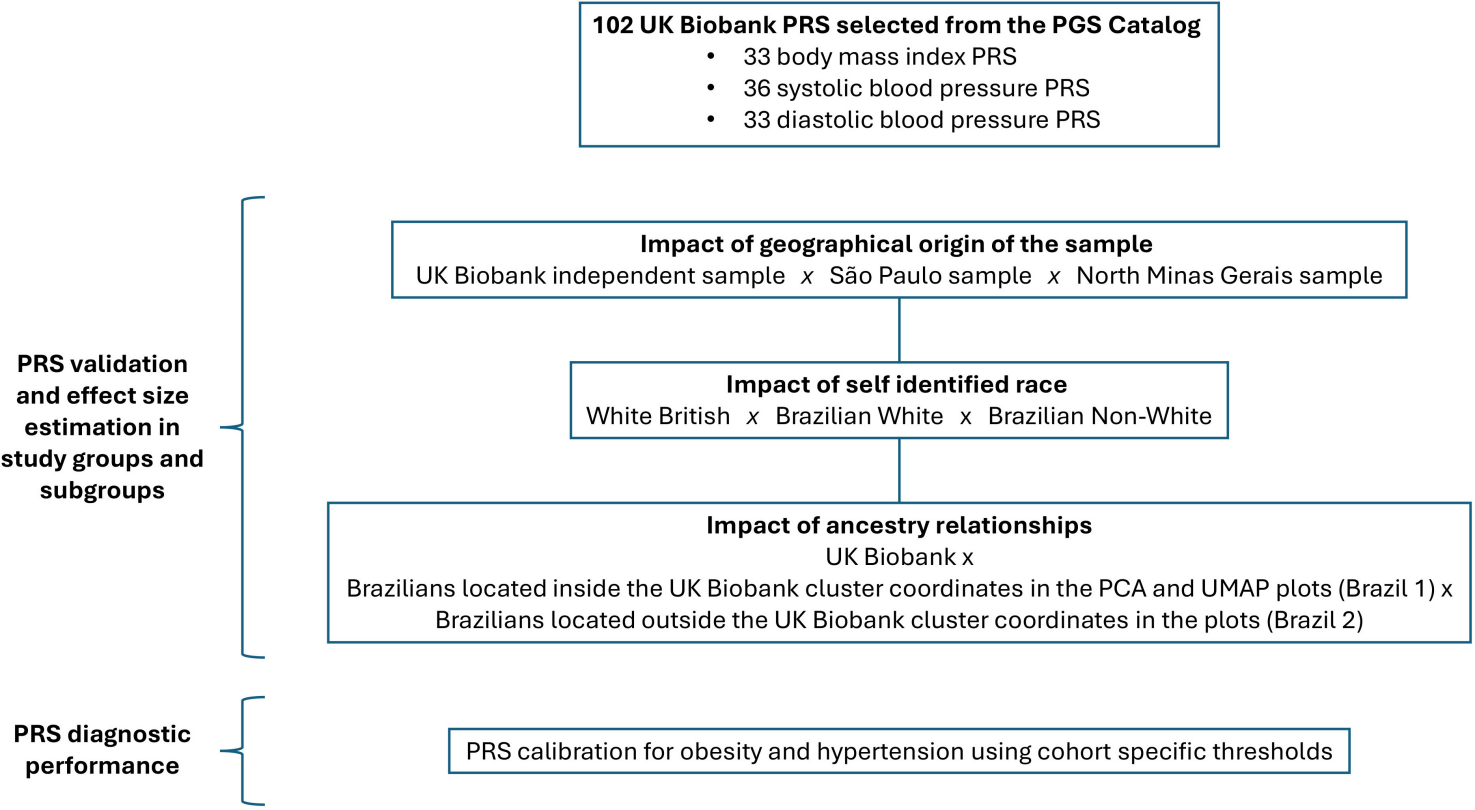
Study design. This study includes 102 publicly available body mass index and blood pressure polygenic scores derived and/or validated with UKB data. Scores were computed in an UKB White British sample and in two Brazilian cohorts from São Paulo and North Minas Gerais. PRS validation and effect size estimation was performed in the study sample populations, in self-identified race subgroups and in UMAP/PCA ancestry relationship subgroups. PRS diagnostic performance was evaluated using cohort specific score thresholds for obesity and hypertension.

Here we investigate the portability of publicly available polygenic risk scores derived from European ancestry populations to the admixed Brazilian population. For this purpose, we examine quantitative traits with well-established polygenic inheritance in humans, namely BMI and blood pressure (13–16). Focusing on scores derived from UKB data, we compare PRS performance in a White British population and in two Brazilian cohorts, one from São Paulo, an urban high-income area, and the other from North Minas Gerais, a rural and economically disadvantaged region. Additionally, we analyze the impact of self-identified race and genetic ancestry on PRS performance (Figure 1).

## 2 Materials and methods

### 2.1 Study population and databases

This study draws upon data from participants enrolled in three major cohort studies: the UKB, the São Paulo-Minas Gerais Tropical Medicine Research Center (SaMi-Trop) research project, and the Brazilian Longitudinal Study of Adult Health (ELSA-Brasil). These studies served as the source for the groups included in the analysis, named the “UKB,” the “North Minas Gerais” and the “São Paulo” samples, respectively. The “North Minas Gerais” and the “São Paulo” samples are termed collectively the “Brazilian samples.”

The UKB project is a large prospective cohort study, in which a wide variety of phenotypic information as well as biological samples have been collected for approximately 500,000 individuals from across the United Kingdom, aged between 40 and 69 at recruitment (17). The main recruitment phase began in April 2007 and was completed in July 2010. The UKB provides an open-access resource available for researchers upon application. In this study, the UKB sample includes 1,586 randomly selected participants of White British ancestry with available baseline anthropometric and genotype data.

SaMi-Trop is a prospective cohort aimed at developing research on Chagas disease. The SaMi-Trop study design and cohort profile have been published previously (18, 19). It enrolled 3.398 individuals seropositive for *Trypanosoma cruzi* infection, with or without cardiac abnormalities. Participants were recruited in 21 municipalities of the northern part of Minas Gerais State in Brazil, between 2013 and 2019. The North Minas Gerais sample includes 1,586 SaMi-Trop participants with available anthropometric and genotype information.

ELSA-Brasil was established as a prospective cohort aimed at investigating cardiovascular diseases. The ELSA-Brasil study design and cohort profile have been published previously (20, 21). Briefly, ELSA-Brasil enrolled 15,105 civil servants from 5 universities and 1 research institute, living in six large Brazilian urban areas (São Paulo, Belo Horizonte, Porto Alegre, Rio de Janeiro, Salvador and Vitória), aged between 35 and 74 years at baseline. The baseline assessment was conducted in 2008–2010 and a follow-up examination took place in 2012–2014. The São Paulo sample includes 1,586 participants enrolled in the São Paulo site with available anthropometric data and genotype information.

### 2.2 Ethics statement

All participants enrolled in the UKB, ELSA-Brasil and SaMi-Trop studies have signed written informed consent for health-related research using the data collected, including biological sample collection, storage and DNA analysis. Both ELSA-Brasil and SaMi-Trop have received approval by the Brazilian National Commission of Ethics in Research (Comissão Nacional de Ética em Pesquisa, CONEP). The present study was approved by the local Ethics Committee of the Hospital das Clínicas, University of São Paulo, in accordance with the Declaration of Helsinki (CAAE: 66201122.6.0000.0068).

### 2.3 Traits and phenotypes

In all samples, weight and standing height were measured using standard equipment and techniques (22). In the present study, body mass index (BMI) is defined as the body weight divided by the square of the body height. Obesity is defined as BMI ≥ 30 kg/m^2^.

In the UKB sample, systolic and diastolic blood pressure were obtained with an electronic blood pressure monitor (Omron 705 IT, OMRON Healthcare Europe B.V., Hoofddorp, Netherlands) by two automated measurements taken in the seated position on the left arm (Data-Fields 4080 and 4079–UKB data resource). The average of the two automated blood pressure recordings was used for analysis. If only one measurement was available, it was taken as the singular value. In the São Paulo sample, resting blood pressure was measured 3 times in the seated position after 5 min’ rest using an oscillometric sphygmomanometer (Omron 765CP; Omron, Kyoto, Japan). The average of the second and third measurements was used in the present analyses (20). For the North Minas Gerais sample, blood pressure was obtained from measurements taken during the second follow-up visit of the SaMi-Trop project.

The definition of hypertension varied across the original cohorts. In the UKB, hypertension was ascertained based on record of a hypertension diagnosis in the available health databases or self-report during interview (23). In ELSA-Brasil, hypertension was defined as systolic blood pressure (SBP) ≥ 140 mmHg or diastolic blood pressure (DBP) ≥ 90 mmHg, or verified treatment with anti-hypertensive medication during the past 2 weeks (21). In SaMi-Trop, hypertension was defined by self-report of a previous diagnosis given by a medical doctor in the past 2 years, as assessed by interview (24). To incorporate these different definitions, hypertension is defined the present study as: (i) self-report of a previous diagnosis given by a medical doctor, (ii) SBP ≥ 140 mmHg, (iii) DBP ≥ 90 mmHg, or (iv) treatment with anti-hypertensive medication.

### 2.4 Whole genome genotyping and imputation

Genotyping of UKB participants involved two methodologies (25). Most participants (438,427) were genotyped using the Applied Biosystems UKB Axiom Array (825,927 markers). A subset of 49,950 participants involved in the UKB Lung Exome Variant Evaluation (UK BiLEVE) study were genotyped using the Applied Biosystems UK BiLEVE Axiom Array by Affymetrix (26). Affymetrix UK BiLEVE Axiom^®^ Array has > 95% content overlap with the UKB Axiom^®^ Array. Affymetrix applied a custom genotype calling pipeline and quality filtering optimized for biobank scale genotyping experiments and the novel genotyping arrays (17).

Blood samples from the São Paulo and Minas Gerais groups underwent DNA extraction and genotyping in the Genetics Laboratory at the Heart Institute, University of São Paulo. Purified DNA was obtained from participants’ peripheral blood using the QIAamp DNA Mini-kit^®^ (27, 28). ELSA-Brasil DNA samples were genotyped using Axiom_PMRA.r3 array (ThermoFisher) and genotypes annotated using the Axiom_PMRA.na35.annot.db provided at the ThermoFisher site. Genotype calling was performed using Affymetrix Power Tools (29). Initial VCF file containing 850,483 variants fulfilled all quality criteria. SaMi-Trop DNA samples were genotyped using two different genotyping arrays: Axiom_PMRA.r3 array (*N* = 2,606) or the Axiom_sarscov array (*N* = 792) (ThermoFisher, Waltham, United States) and genotypes annotated using the array specific annotation file provided at the ThermoFisher website. Genotype calling was performed using Affymetrix Power Tools. The initial VCF file contained 701,985 (for the PMRA array) and 803,863 (for the sarscov array) variants before quality control filtering.

To account for differences in genotyping arrays and avoid bias in PRS analysis introduced by uneven availability of genetic makers across samples, imputation was conducted simultaneously in the three study populations. Markers were retained only if available in all three samples. Imputation was performed with the Haplotype Reference Consortium Michigan Imputation Server using the TOPMED reference haplotype panel (for mixed samples) as reference (30, 31). After imputation, data were exported in the standard PLINK format, downstream quality control procedures and statistical analysis were conducted using the latest PLINK and R software packages, installed on a Linux-based computation resource. Imputation markers were kept if *R*^2^ > 0.3, and minor allele frequency (MAF) > 0.01. A Hardy-Weinberg equilibrium (HWE) *p-*value < 1 × 10^–20^ was used to control for potential genotyping clustering problems.

### 2.5 Genetic ancestry analysis

Population genetic ancestry relationship was inferred using Uniform Manifold Approximation and Projection (UMAP) and Principal Component Analysis (PCA). UMAP and PCA were applied to the combined study samples to enable spatial visualization of similar genetic background across study participants (32, 33). Plots were constructed using PLINK (v. 1.9) and R software (v. 4.4.3) packages.

### 2.6 Self-identified race

Self-identified race in the Brazilian samples follows the classification used in censuses conducted by the Brazilian Institute of Geography and Statistics (Instituto Brasileiro de Geografia e Estatística, IBGE), which includes five categories: “Branco” (White), “Pardo” (Multiracial/Mixed), “Preto” (Black), “Amarelo/Asiático” (Yellow/Asian), and “Indígena” (Indigenous). Individuals were asked to self-identify within these categories. To allow analysis of the potential influence of self-identified race in PRS performance, the São Paulo and North Minas Gerais samples were divided into the “Brazilian White” and “Brazilian Non-White” subgroups. The UKB sample included only individuals who self-identified as White British (Data-Field 21000, Ethnic background—UKB data resource).

### 2.7 Polygenic risk score selection and computation

PRS were selected based on the following criteria: (i) being specifically designed for BMI, SBP or DBP, and (ii) developed using genome-wide association study (GWAS) summary statistics, training and/or validation cohorts from the UKB. A total of 102 scores were included: 33 for BMI, 36 for SBP, and 33 for DBP. PRS information was obtained from the PGS Catalog (version 2024-01-26), an online resource providing complete data for score computation (34). The scores’ main features, including number of variants, development methods and original performance metrics are described in Supplementary Table 1.

PRS were computed using PLINK. To ensure consistent marker inclusion across samples, scores were calculated jointly for all participants from the three study groups. To enable cross-score comparison, the individual computed PRS values were standardized to z-score units. Score transformation used value distribution across the entire study population to preserve comparability across study groups (35).

### 2.8 Statistical analysis

Participant baseline characteristics were summarized using descriptive statistics. Continuous phenotypes were described as medians and interquartile ranges and compared using Kruskal-Wallis followed by Dunn test. Categorical phenotypes were described as counts and percentage and compared using Chi-square test.

PRS validation in the study samples and analysis of their performance followed a stepwise approach, described below. Score validation refers to the finding of a statistically significant association between a score and a trait in a specific sample group as assessed by a regression model. PRS performance refers to the effect size of a previously validated association. PRS coefficients in regression models (*betas*) were chosen as a performance metric. Once PRS raw values were standardized to z-score units, *betas* reflect the change in the trait per one standard deviation increase in the PRS. All statistical analysis were conducted with R software (version 4.4.3).

#### 2.8.1 PRS validation in the study samples and evaluation of geographic location effects

The association between each PRS and its corresponding trait (BMI, SBP, or DBP) was tested in each sample using linear regression models. Traits were treated as continuous variables. Multivariable models included age, sex, and the first four PCs as covariates (based on a screen plot analysis of the eigenvalues for the 10 first PC). PRS were considered validated if significantly associated with the respective trait after false discovery rate (FDR) correction (adjusted *p* < 0.05). Linear regression coefficients (*betas*) were used to quantify effect sizes. A mixed effects model was built to assess the differences in the overall ability of PRS to associate with the traits across the three geographically separate samples (Equation 1). Self-identified race, ancestry, age and sex were included as covariates. The ability of PRS to associate with the traits was considered to differ between two given samples at a statistically significant threshold of *p* < 0.001.

Equation 1—Mixed effects model for evaluation of geographic location effects on PRS association with the traits.


$$T\,r\,a\,i\,t∼P\,R\,S_{v\,a\,l\,u\,e}+R\,e\,g\,i\,o\,n+R\,a\,c\,e+A\,n\,c\,e\,s\,t\,r\,y+A\,g\,e+S\,e\,x+$$

$$P\,R\,S_{v\,a\,l\,u\,e}.R\,e\,g\,i\,o\,n+\left(1|P\,R\,S\,\_\,I\,D\right)$$

Trait refers to body mass index, systolic or diastolic blood pressure. PRS_*value*_ refers to the computed PRS individual value standardized to z-score units. Region refers to geographical origin of the sample (United Kingdom, São Paulo or North Minas Gerais). Race refers to self-identified race (White British, Brazilian White or Brazilian Non-White). Ancestry refers to ancestry group as defined by overlap with the British in the UMAP and PCA plots (UKB, subgroups Brazil 1 or Brazil 2). PRS_ID refers to the individual identification of the score.

The North Minas Gerais sample included individuals seropositive for *T. cruzi* and thus potentially affected with Chagas cardiomyopathy. To control for the impact of heart failure on BMI and blood pressure phenotypes, sensitivity analyses were performed after the previously described statistical tests, excluding individuals with a left ventricular ejection fraction (LVEF) < 50%.

#### 2.8.2 Evaluation of self-identified race and genetic ancestry effects

Two subgroup analyses were performed to assess PRS–trait associations within prespecified groups. The first analysis evaluated the influence of self-identified race on PRS validation and performance. This analysis included the following groups: (i) White British individuals, who make up the entire UKB sample, (ii) self-identified White individuals from São Paulo and North Minas Gerais, and (iii) self-identified Non-White individuals from São Paulo and North Minas Gerais. The second analysis assessed the impact of genetic ancestry, comparing PRS validation and performance across the UKB and two Brazilian ancestry subgroups. Taking as reference the UKB sample coordinates in the UMAP and PCA plots, the Brazilian samples were divided into the following subgroups: (i) Brazilians in spatial overlap with the UKB population on the plots, thus inferring genetic similarity (Brazil 1), and (ii) Brazilians not overlapping with the UKB sample (Brazil 2).

Multivariable linear models, adjusted for age and sex, were applied to continuous traits (BMI, SBP, and DBP). PRS were considered validated in the subgroups if significantly associated with the respective trait after false discovery rate (FDR) correction (adjusted *p* < 0.05). Linear regression coefficients (betas) were used to quantify effect sizes. Mixed effects models were built to assess the differences in the overall ability of PRS to associate with the traits across the self-identified race groups (Equation 2) and ancestry groups (Equation 3). The ability of PRS to associate with the traits was considered to differ between two given subgroups at a statistically significant threshold of *p* < 0.001.

Equation 2—Mixed effects model for evaluation of self-identified race effects on PRS association with the traits.


$$T\,r\,a\,i\,t∼P\,R\,S_{v\,a\,l\,u\,e}+R\,e\,g\,i\,o\,n+R\,a\,c\,e+A\,n\,c\,e\,s\,t\,r\,y+A\,g\,e+S\,e\,x+$$

$$P\,R\,S_{v\,a\,l\,u\,e}.R\,a\,c\,e+\left(1|P\,R\,S\,\_\,I\,D\right)$$

Equation 3—Mixed effects model for evaluation of ancestry effects on PRS association with the traits.


$$T\,r\,a\,i\,t∼P\,R\,S_{v\,a\,l\,u\,e}+R\,e\,g\,i\,o\,n+R\,a\,c\,e+A\,n\,c\,e\,s\,t\,r\,y+A\,g\,e+S\,e\,x+$$

$$P\,R\,S_{v\,a\,l\,u\,e}.A\,n\,c\,e\,s\,t\,r\,y+\left(1|P\,R\,S\,\_\,I\,D\right)$$

The terms in Equations 2, 3 are defined as in Equation 1.

#### 2.8.3 Evaluation of PRS diagnostic performance

Irrespective of their association with continuous traits (BMI, SBP, or DBP), PRS may still associate with related clinical diagnoses, such as obesity or hypertension. These associations could be enhanced by applying diagnostic thresholds tailored to each study sample. To evaluate the ability of PRS to classify individuals with obesity or hypertension, multivariable logistic regression models were built. Models were adjusted for age, sex, and the first four PCs. In each study sample, PRS distributions were divided into quintiles, defining risk categories specific to each sample. Classification performance was assessed by comparing individuals in the highest (5th) risk quintile to those in the lowest (1st) quintile, with odds ratios (OR) as the performance metric. After that, a single PRS with strong classification performance in the quintile-based analysis was selected for each trait. Samples were randomly split into training and test sets. Univariate logistic regression models were fitted, and the precision–recall curve, F1-score, and optimal threshold were derived from the training data. Confusion matrix metrics and the area under the precision–recall curve (AUPRC) were computed in the test set. To account for the potential bias introduced by arbitrarily splitting the samples, the procedure was repeated 100 times; results represent the mean across iterations.

## 3 Results

### 3.1 Study population baseline characteristics

The three study samples included mainly women (55.4, 54.9, and 67.2% in the UKB, São Paulo and North Minas Gerais samples, respectively) and middle-aged participants (median age 59, 51, and 58 years in the UKB, São Paulo and North Minas Gerais samples, respectively). Samples differed across several characteristics. North Minas Gerais had a higher prevalence of hypertension (62.6% vs. 31.7 and 23.7% in the São Paulo and UKB samples, respectively) and a lower prevalence of obesity (17.9vs. 24.3 and 23.6% in the São Paulo and UKB samples, respectively). Additionally, the two Brazilian samples differed in self-identified race composition. There was a predominance of Mixed in the North Minas Gerais sample, while a higher proportion of Whites was found in the São Paulo sample. The North Minas Gerais sample included 1114 (70.24%) Chagas cardiomyopathy cases, as defined by the presence of major ECG abnormalities. However, the mean LVEF was 60.9%, and only 203 participants (12.8%) exhibited LVEF < 50%. Study population baseline characteristics are summarized in Supplementary Table 2.

### 3.2 Genetic architecture of the study population and ancestry relationships

**FIGURE 2:**
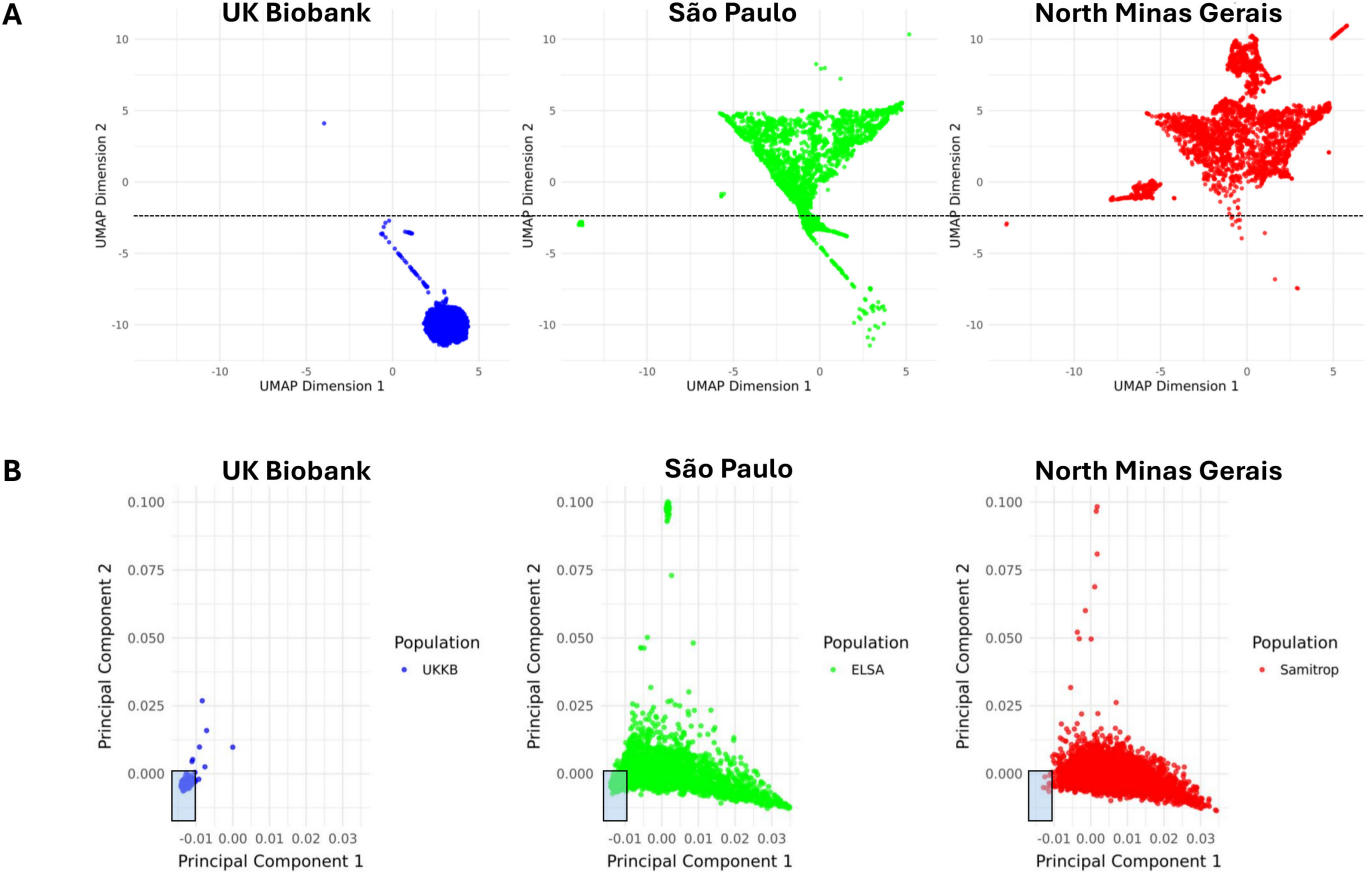
UMAP and PCA plots of study population genotypes. **(A)** UMAP plots. Dimension 1 is shown on the x axis and dimension 2 is shown on the y axis. The black dotted line indicates the threshold of –2.5 on the y axis, under which 99.9% of the UKB population is located. **(B)** PCA plots. PC1 is shown on the x axis and PC2 is shown on the y axis. The shaded rectangles indicate the area in which 99.4% of the UKB population is located and the overlapping individuals in the Brazilian samples. PCA, principal component analysis. UKB, United Kingdom Biobank. UMAP, uniform manifold approximation and projection.

Study population genomic structure was studied with UMAP and PCA (Figure 2). UMAP and PCA analyses show the UKB sample as a dense cluster, whereas the Brazilian samples exhibit a considerably more dispersed distribution across the embedding space. In the UMAP plots, 1,585 British individuals (99.9%) cluster below dimension 2 < − 2.5 but only 404 Brazilian subjects (12.7%) are contained within this threshold. Similarly, the area within PC1 < - 0.01 and PC2 < 0.00 contains 1576 British individuals (99.4%), while only 390 Brazilian participants (12.3%) overlapped with UKB participants in the same area. Most Brazilian individuals overlapping with the UKB belong to the São Paulo sample (99.3 and 98.2% in the UMAP and PCA plots, respectively). This indicates that the Brazilian samples have a distinct genetic ancestry composition and hold more genetic diversity when compared to the UK sample.

We examined the accuracy of Brazilian White self-identified race as a marker of genetic ancestry similarity to the UKB. Brazilians’ self-identification as White was significantly associated to localization within the UKB cluster coordinates by PCA and UMAP analysis (chi-squared test *p* < 0.001). In fact, most Brazilian subjects overlapping with UKB individuals in the UMAP and PCA plots self-identify as White (79.7 and 93.8%, respectively). Nevertheless, out of a total 1241 Brazilians who self-identified as White in the combined Brazilian samples, only 29.5 and 25.9% overlapped with the British by PCA and UMAP analysis, respectively. Overlap of self-identified Whites with the UKB was lower in the North Minas Gerais sample, with only 1.2% and 0.9% of Whites overlapping with the UKB by PCA and UMAP analysis, respectively. Self-identification as White in the Brazilians samples had good sensitivity for identification of European ancestry (80% and 96% in UMAP and PCA, respectively) and a good negative predictive value (96 and 99%). However, specificity was only moderate (67% and 70% in UMAP and PCA, respectively) and positive predictive value was low (26 and 34%).

### 3.3 Validation and performance of UKB based PRS in the Brazilian samples and subgroups

The number of polygenic scores significantly associated with the respective trait in the study samples and subgroups is shown in Table 1. The estimated overall PRS effect sizes in the study samples and subgroups are presented in Table 2.

**TABLE 1:** Polygenic scores validated in the study samples and subgroups.

Geographical origin of the sample			
PRS	UKB (*n* = 1,586)	São Paulo (n = 1,586)	North Minas Gerais (n = 1,586)
BMI (*n* = 33)	33 (100)	32 (96.7)	30 (90.9)
SBP (*n* = 36)	35 (97.2)	24 (66.7)	14 (38.9)
DBP (*n* = 33)	31 (93.9)	23 (69.7)	18 (54.5)
**Self-identified race group**			
**PRS**	**White British (*n* = 1,586)**	**Brazilian White** **(*n* = 1,241)**	**Brazilian Non-White** ** (*n* = 1,910)**
BMI (*n* = 33)	33 (100)	31 (93.9)	31 (93.9)
SBP (*n* = 36)	35 (97.2)	15 (41.6)	22 (61.1)
DBP (*n* = 33)	31 (93.9)	26 (78.8)	21 (63.6)
**Genetic ancestry relationship—UMAP**			
**PRS**	**UKB (*n* = 1,586)**	**Brazil 1 (*n* = 404)**	**Brazil 2 (*n* = 2,768)**
BMI (*n* = 33)	33 (100)	30 (90.9)	32 (96.7)
SBP (*n* = 36)	35 (97.2)	18 (50.0)	24 (66.7)
DBP (*n* = 33)	31 (93.9)	20 (60.6)	24 (72.7)
**Genetic ancestry relationship—PCA**			
**PRS**	**UKB (*n* = 1,586)**	**Brazil 1 (*n* = 390)**	**Brazil 2 (*n* = 2,782)**
BMI (*n* = 33)	33 (100)	32 (96.7)	32 (96.7)
SBP (*n* = 36)	35 (97.2)	16 (44.4)	25 (69.4)
DBP (*n* = 33)	31 (93.9)	19 (57.6)	23 (69.7)

Number of PRS associated with the corresponding trait in the multivariable linear regression model in the study samples (adjusted *p*< 0.05) and subgroups (unadjusted *p* < 0.05). Brazil 1 is the subgroup of Brazilians overlapping with the UKB on PCA and UMAP plots. Brazil 2 is the subgroup of Brazilians located outside the area contained by the UKB cluster coordinates in the plots. BMI, body mass index. DBP, diastolic blood pressure. PCA, principal component analysis. PRS, polygenic risk score. SBP, systolic blood pressure. UMAP, Uniform Manifold Approximation and Projection. UKB, United Kingdom Biobank.

**TABLE 2:** Overall PRS effect sizes in the study samples and subgroups.

Geographical origin of the sample			
Trait	UKB (*n* = 1,586)	São Paulo (*n* = 1,586)	North Minas Gerais (*n* = 1,586)
BMI	+ 1.75 (*p* < 0.001)	+ 1.06	+ 0.67 (*p* < 0.001)
SBP	+ 4.56 (*p* < 0.001)	+ 1.11	+ 1.44 (*p* = 0.007)
DBP	+ 2.31 (*p* < 0.001)	+ 0.96	+ 0.78 (*p* = 0.023)
**Self-identified race group**			
**Trait**	**White British (*n* = 1,586)**	**Brazilian White** **(*n* = 1,241)**	**Brazilian Non-White** **(*n* = 1,910)**
BMI	+ 1.76 (*p* < 0.001)	+ 1.24 (*p* < 0.001)	+ 0.64
SBP	+ 4.56 (*p* < 0.001)	+ 1.21 (*p* = 0.704)	+ 1.26
DBP	+ 2.31 (*p* < 0.001)	+ 1.00 (*p* = 0.018)	+ 0.82
**Genetic ancestry relationship—UMAP**			
**Trait**	**UKB (*n* = 1,586)**	**Brazil 1 (*n* = 404)**	**Brazil 2 (*n* = 2,768)**
BMI	+ 1.75 (*p* < 0.001)	+ 1.22	+ 0.83 (*p* < 0.001)
SBP	+ 4.56 (*p* < 0.001)	+ 1.36	+ 1.22 (*p* = 0.424)
DBP	+ 2.31 (*p* < 0.001)	+ 0.97	+ 0.88 (*p* = 0.364)
**Genetic ancestry relationship—PCA**			
**Trait**	**UKB (*n* = 1,586)**	**Brazil 1 (*n* = 390)**	**Brazil 2 (*n* = 2,782)**
BMI	+ 1.75 (*p* < 0.001)	+ 1.36	+ 0.83 (*p* < 0.001)
SBP	+ 4.56 (*p* < 0.001)	+ 1.35	+ 1.20 (*p* = 0.389)
DBP	+ 2.31 (*p* < 0.001)	+ 1.18	+ 0.83 (*p* < 0.001)

Comparison across PRS effect size in the study groups was performed with a multivariable mixed effects model. BMI PRS betas reflect changes in BMI measured in kg/m2 per SD change in the PRS. Blood pressure PRS betas reflect changes in systolic or diastolic blood pressure measured in mmHg per SD change in the PRS. Brazil 1 is the subgroup of Brazilians overlapping with the UKB on PCA and UMAP plots. Brazil 2 is the subgroup of Brazilians located outside the area contained by the UKB cluster coordinates in the plots. BMI, body mass index. DBP, diastolic blood pressure. PCA, principal component analysis. PRS, polygenic risk score. SBP, systolic blood pressure. UMAP, Uniform Manifold Approximation and Projection. UKB, United Kingdom Biobank.

#### 3.3.1 Impact of geographical origin of the sample on PRS performance

**FIGURE 3:**
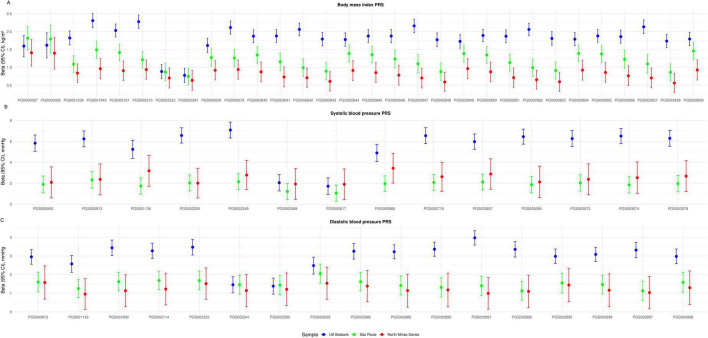
Effect size of polygenic risk scores validated in the UKB, São Paulo and North Minas Gerais samples. **(A)** Thirty validated BMI polygenic scores. **(B)** Fourteen validated SBP scores. **(C)** Seventeen validated DBP scores. Dots represent the linear regression coefficients (betas). The vertical bars correspond to the 95% confidence intervals. PRS raw values were standardized to z-score units; betas reflect the change in the outcome per one standard deviation increase in the PRS. BMI, body mass index. CI, confidence interval. DPB, diastolic blood pressure. PRS, polygenic risk score. SBP, systolic blood pressure. UKB, United Kingdom Biobank.

The proportion of polygenic scores significantly associated with the respective traits was higher in the UKB sample and lower in the North Minas Gerais sample. Scores for BMI had a higher rate of validation in all samples in comparison to blood pressure PRS. Figure 3 shows the linear regression coefficients of PRS validated in the three geographically separated samples. The overall PRS effect size was compared across samples, taking São Paulo as the reference group. BMI PRS effect is significantly higher in the UKB than in São Paulo by +0.69 kg/m^2^ per SD of PRS (*p* < 0.001) and lower in North Minas Gerais by -0.39 kg/m^2^ per SD of PRS (*p* < 0.001). While SBP PRS effect is significantly higher in the UKB than in São Paulo by +3.45 mmHg per SD of PRS (*p* < 0.001), there is no significant difference in SBP PRS effect between São Paulo and North Minas Gerais (*p* = 0.007). Similarly, DBP PRS effect is higher in the UKB than in São Paulo by +1.35 mmHg per SD of PRS (*p* < 0.001), but there is no difference in DBP PRS effect size between São Paulo and North Minas Gerais (*p* = 0.023). Thus, a pattern emerges in which PRS betas are higher in the UKB and lower in the Brazilian samples. Comparing the two Brazilian samples, BMI PRS have higher effect size in the São Paulo sample, while blood pressure PRS have a comparable effect size in the two groups. Sensitivity analysis excluding individuals with LVEF < 50% from the North Minas Gerais sample yielded identical results.

#### 3.3.2 Impact of self-identified race on PRS performance

Brazilian participants from the São Paulo and North Minas Gerais samples were divided into the “Brazilian White” (*n* = 1241) and “Brazilian Non-White” (*n* = 1910) subgroups for analysis of the potential influence of self-identified race on PRS performance. Across all traits, the two Brazilian self-identified race groups had lower PRS validation rates than the White British. Validation rates were identical for BMI PRS in the Brazilian Whites and Non-Whites, higher in Brazilian Non-Whites for SBP PRS and in Brazilian Whites for DBP PRS. Supplementary Figure 1 shows the linear regression coefficients of PRS validated in the UKB and in the Brazilian self-identified race subgroups. The overall PRS effect size was compared across subgroups, taking the Brazilian Non-Whites as the reference group. BMI PRS effect size is significantly higher in the UKB than in the Brazilian Non-Whites by +1.12 kg/m^2^ per SD of PRS (*p* < 0.001); it is also higher in Brazilian Whites than in Non-Whites by + 0.60 kg/m^2^ per SD of PRS (*p* < 0.001). While SBP PRS effect size is significantly higher in the UKB than in the Brazilian Non-White by +3.30 mmHg per SD of PRS (*p* < 0.001), there is no significant difference in SBP PRS effect between the two Brazilian self-identified race subgroups (*p* = 0.704). Similarly, DBP PRS effect size is significantly higher in the UKB than in the Non-Whites by +1.49 mmHg per SD of PRS (p < 0.001), but there is no difference in DBP PRS effect between Brazilian Whites and Non-Whites (*p* = 0.018). Therefore, PRS betas remain higher in the UKB than in both Brazilian self-identified race subgroups. BMI PRS betas are higher in Brazilian Whites than in Brazilian Non-Whites, but blood pressure PRS show comparable effect size between the two Brazilian subgroups.

#### 3.3.3 Impact of genetic ancestry similarity on PRS performance

Taking as reference the UKB cluster coordinates on the plots, the Brazilian participants were split into two subgroups. Subgroup Brazil 1 includes Brazilians sharing genetic ancestry similar to the British, inferred by spatial overlap with the UKB sample on the plots. In the UMAP analysis, this group is defined by location within UMAP dimension 2 ≤ -2.5 (*n* = 404). In the PCA analysis, it is delimited by PC1 ≤ -0.01 and PC2 ≤ 0.00 (*n* = 390). Subgroup Brazil 2 comprises Brazilian participants genetically distant to the UK sample. In the UMAP analysis, Brazil 2 was defined by location beyond UMAP dimension 2 > -2.5 (*n* = 2,768). In the PCA analysis, it was defined by PC1 > -0.01 or PC2 > 0.00 (*n* = 2,782).

Across all traits, PRS validation rates are higher in the UKB group, but the subgroup of Brazilians genetically similar to the British (Brazil 1) failed to show improved score validation rates when compared to subgroup Brazil 2. Linear regression coefficients of validated PRS are shown in Supplementary Figures 2, 3 for the UMAP and PCA subgroups, respectively. The overall PRS effect size was compared across subgroups, taking Brazil 1 as the reference group. BMI PRS effect is significantly higher in the UKB than in Brazil 1 by +0.53 and by +0.39 kg/m2 per SD of PRS in UMAP and PCA analysis, respectively (*p* < 0.001 for both); it is also higher in Brazil 1 than in Brazil 2 by +0.39 and by +0.53 kg/m^2^ per SD of PRS in UMAP and PCA analysis, respectively (*p* < 0.001 for both). While SBP PRS effect is significantly higher in the UKB than in Brazil 1 by +3.2 mmHg per SD of PRS in UMAP and PCA analysis (*p* < 0.001 for both), there is no significant difference in SBP PRS effect between the two Brazilian ancestry subgroups (*p* = 0.424 and 0.389, respectively). DBP PRS effect is significantly higher in the UKB than in Brazil 1 by +1.34 and by 1.13 mmHg per SD of PRS in UMAP and PCA, respectively (*p* < 0.001 for both). While DBP PRS effect size was equivalent in both Brazilian subgroups by UMAP analysis, it was higher in Brazil 1 than in Brazil 2 in PCA (*p* < 0.001). Therefore, PRS effect sizes were higher in the UKB than in the Brazilian ancestry subgroups. BMI scores present higher effect size in the subgroup of Brazilians genetically similar to the UKB population than amongst those who are distant from the British. However, blood pressure PRS show comparable effect size across the two Brazilian subgroups.

### 3.4 PRS diagnostic performance

To evaluate the ability of PRS to classify individuals with obesity or hypertension, PRS distributions were divided into quintiles, defining risk categories specific to each sample. Classification performance was assessed by comparing individuals in the highest (5th) risk quintile to those in the lowest (1st) quintile. The number of scores significantly associated to the diagnosis in all three samples was as follows: 29 (87.9%) BMI PRS, 15 (41.7%) SBP PRS and 15 (45.5%) DBP PRS. Across all traits, the OR for diagnosis among individuals in the highest quintile of polygenic risk was higher in the UKB sample, but comparable between the São Paulo and Minas Gerais samples. The OR of validated PRS are show in Supplementary Figure 4.

To further explore the possibility of enhancing PRS diagnostic performance, optimal sample-specific thresholds for diagnosis were derived from the precision-recall curve F1-scores. The following previously validated PRS were selected for testing: PGS002842 (body mass index), PGS002807 (systolic blood pressure) and PGS002639 (diastolic blood pressure). In general, scores had poor accuracy for obesity and hypertension. For PGS002842, an area under the precision-recall curve (AUPRC) of 0.53 was found in the UKB but it was only 0.26 in the North Minas Gerais sample. For blood pressure PRS, an AUPRC of 0.68 was found in the North Minas Gerais sample but it was only 0.37 for the other samples. Score sensitivity, specificity, positive and negative predictive values and AUPRC are shown in Supplementary Table 3.

## 4 Discussion

### 4.1 Limited reproducibility of UKB polygenic scores in Brazilians and trait-dependent heterogeneity

Not all UKB-based PRS replicated in Brazilian cohorts, and those that did generally showed attenuated effect sizes. BMI PRS displayed the highest reproducibility, with over 90% validated in both Brazilian samples. In contrast, validation rates for systolic and diastolic blood pressure PRS were markedly lower. For example, only 66.7% of SBP PRS were validated in the São Paulo cohort, and just 38.9% in the North Minas Gerais cohort. Effect sizes were consistently weaker in the Brazilian cohorts than in the UKB, as confirmed by mixed-effects models.

Such comparison across study samples considered the PRS value distribution in the entire study population. We also analyzed PRS performance by independently considering score value distribution in each sample. Nevertheless, risk stratification based on sample-specific PRS quintiles produced much lower OR for obesity and hypertension in Brazilian samples than in the UKB. Similarly, selected PRS demonstrated poor accuracy for both traits across all cohorts despite sample-specific thresholds optimized via F1-scores.

These results corroborate prior findings. A BMI PRS developed using an European-ancestry GWAS and trans-ethnic training data has been previously validated in the ELSA-Brasil cohort, albeit with reduced predictive performance (9). In another study, a SBP PRS was derived from UKB data and evaluated in two Brazilian cohorts, showing significant associations with SBP and hypertension, but with weaker effects in Brazilians and lack of association with stage 1 hypertension in one of the cohorts (10).

Several factors may explain the trait-specific differences observed in PRS validation rates. Environmental exposures are likely to contribute similarly to BMI variability in Brazil and in the UK, whereas their influence may be more pronounced for blood pressure phenotypes in Brazilian populations. In addition, anthropometric measures such as height and weight are less prone to measurement error and short-term variability than blood pressure, leading to more reliable BMI phenotyping. Importantly, while score validation rates may be high for some traits, for others many scores fail to replicate and, in any case, validated scores yield weaker effects. Calibration of PRS in Brazilian samples did not result in meaningful performance improvements. Overall, these findings indicate that UKB–derived PRS are not directly transferable to Brazilian populations and emphasize the need for empirical validation in Brazilian cohorts prior to their application in research or clinical practice.

### 4.2 Differences in PRS performance across Brazilian regions are of small magnitude

The São Paulo and North Minas Gerais cohorts represent populations from distinct geographic regions with diverse social, economic, and cultural characteristics. A comparable proportion of BMI PRS were validated in both regions, although validated scores showed stronger effect sizes in São Paulo. Blood pressure PRS behaved differently: validation rates for SBP and DBP were higher in São Paulo than in North Minas Gerais, but the effect sizes of validated scores were similar across regions. Overall, these results suggest a slightly better performance of UKB-based PRS in São Paulo as compared to North Minas Gerais. This in line with the findings of Teixeira et al., who also reported small interregional differences in performance of a SBP PRS in Brazil (10). It must be noted, however, that differences between São Paulo and North Minas Gerais were minor compared to the much larger gap between Brazilian cohorts and the UKB. In fact, for all traits, the UKB sample had consistently more validated scores and higher PRS effect sizes.

### 4.3 Self-identified race and genetic ancestry similarity do not modulate European-based PRS performance in Brazilian cohorts

Genetic clustering analyses show that Brazilian samples are substantially more heterogeneous than the UKB sample, as reflected by the wider dispersion of individuals from São Paulo and North Minas Gerais in the UMAP and PCA plots. Such heterogeneity suggests greater interindividual variation, which is expected to reduce PRS accuracy in highly admixed populations (5). If the limited transferability of European-derived scores to Brazilians were primarily driven by differences in ancestry and admixture, PRS performance should improve in subgroups with lower genomic heterogeneity and greater similarity to European reference groups. We tested this assumption using subgroup analyses based on self-identified race and genetic clustering.

In Brazil, self-identified race is often used as a proxy for genetic ancestry under the assumption that individuals identifying as Black have higher African ancestry, while those identifying as White have higher European ancestry. Prior studies support a positive association between Black self-identification and African ancestry (27, 36), whereas the association between White self-identification and European ancestry is weaker and more variable (36). To assess whether White self-identification could still capture a subgroup with higher European ancestry and improved PRS transferability, we evaluated PRS performance across racial categories. However, BMI PRS validation rates were identical between White and Non-White subgroups, though effect sizes were higher in Whites. Conversely, SBP and DBP PRS showed differing validation rates between Whites and Non-Whites, but effect sizes were comparable. Overall, self-identified race did not reliably delineate a Brazilian subgroup with enhanced performance of UKB–derived PRS.

It could be argued that White self-identification does not relate to improved UKB PRS transferability because it lacks specificity as an indicator of European ancestry in Brazilians. Genetic ancestry, understood as genetic similarity of an individual to a reference population, has been proposed as an alternative criterion for predicting PRS accuracy (5). To test this, we examined PRS performance among individuals genetically closest to the British, identified using two clustering approaches (UMAP and PCA). Both approaches produced similar results. Although BMI scores had greater effect size in the subgroup of Brazilians genetically similar to the UKB population, such effect did not match that observed in the UKB sample. Blood pressure PRS presented effect sizes inferior to the UKB group and comparable across the two Brazilian ancestry subgroups. Thus, even genetic ancestry similarity did not identify a Brazilian subgroup with PRS performance equivalent to that observed in the UKB.

### 4.4 Potential reasons for limited performance of UKB PRS in the Brazilian samples

The reduced predictive capability of European-derived PRS in Brazilian samples was previously postulated to stem from their complex genetic composition of European, African, and Native American ancestries, so that the fewer European genetic contributions, the more significant the expected drop in predictive performance (10). In our study, however, European-ancestry inference failed to identify Brazilian individuals with improved PRS transferability. As such, the diminished PRS accuracy observed in Brazilians may be explained by alternative mechanisms. We consider three non-exclusive theoretical explanations: (i) genotype–environment interactions may account for a larger proportion of trait variance in the Brazilian samples; (ii) European-based PRS capture only a limited portion of the underlying genetic architecture of complex traits; and (iii) technical limitations related to genotype imputation in Brazilians hinder accurate PRS computation. These explanations are speculative, and additional data is needed for definitive conclusions.

#### 4.4.1 Genotype–environment interactions may account for a larger share of trait variance in the Brazilian samples

In principle, the narrow-sense heritability of a trait should be consistent across populations, meaning that additive genetic effects explain a fixed proportion of the phenotypic variance. In practice, however, narrow-sense heritability cannot be reliably estimated in human populations due to genotype-environment correlation and the difficulty to design human population studies that effectively control for environmental effects (37). The same trait can be more environmentally driven in one setting than in another. Thus, as a statistical estimate, heritability reflects the specific environmental context in which it is measured.

PRS are designed to capture the additive genetic component of trait variance. When environmental influences represent a larger share of total variance, the proportion attributable to genotype—and thus detectable by PRS—necessarily decreases. It is therefore possible that environmental determinants (e.g., diet, physical activity patterns, socioeconomic exposures, disease burden) contribute more strongly to BMI and blood pressure variation in the Brazilian cohorts than in the UKB. Under such circumstances, UK-derived PRS would be expected to show reduced accuracy because the genotype-driven variation they capture represents a smaller proportion of overall phenotypic variance in the Brazilian samples.

#### 4.4.2 European-derived PRS capture an incomplete fraction of the underlying genetic architecture of complex traits

Causal genetic variants are presumed to be shared by different ancestries (6, 37, 38). However, neither the exact number of causal variants nor their true effect sizes can be reliably estimated (6). Genetic drift and natural selection lead to marked variation in linkage disequilibrium (LD) patterns and allele frequency across populations. When discovery GWAS are restricted to single ancestries, substantial differences in variant identification and effect size estimation are observed across populations because of LD and allele frequency variation (2, 6). This drives reductions in the relative accuracy of PRS across different populations (6).

Causal variants that are rare or absent in European cohorts may remain undetected, even if they are relevant in other populations. Supporting this possibility, GWAS performed on a large Han Chinese population from the Taiwan Precision Medicine Initiative revealed 95 new causal variants across several traits, of which 33 were rare in the European population (MAF < 0.01), explaining why they were not previously reported (39). Similarly, GWAS performed on the Department of Veterans Affairs Million Veteran Program population identified 3477 variant-trait associations which became significant only when individuals from non-European populations were included in the analysis (40). The Brazilian population is expected to harbor several undetected causal variants. An analysis of 2,723 high-coverage whole genomes from Brazil identified 8,721,871 novel SNPs absent from major public datasets, including over 36,000 potentially deleterious variants and more than 2,000 putative loss-of-function (pLOF) variants (8). Interestingly, 222 genes harboring pLOF variants were associated with body mass index (BMI). Therefore, it is possible that some BMI and blood pressure variants are not captured by UKB scores but exert a substantial influence on these traits in the Brazilian population.

Genetic variants included in UKB PRS may have distinct effect sizes in other populations, due to differences in LD patterns and allele frequencies. Admixed populations such as the Brazilian one have complex LD structures, which are largely unmapped and for which reference panels are lacking (41). In fact, the decreased PRS accuracy in Brazilians irrespective of European ancestry could be explained by a deviation from the European LD structure and allele frequency landscape that is not alleviated in population segments more closely resembling European backgrounds. The resulting difference in variant effect size would drive reduced PRS accuracy in Brazilians.

In conclusion, genomic data from Brazilian and other non-European populations should be incorporated in GWAS for effective variant fine-mapping and effect size estimation, enabling optimal PRS accuracy.

#### 4.4.3 Technical limitations related to genotype imputation may influence PRS computation in Brazilian samples

Genotype imputation predicts unobserved genotypes using LD patterns derived from a reference haplotype panel (42). In our study, samples were imputed using the TOPMed reference panel, which incorporates genomic data from diverse ancestries (43). Nonetheless, imputation panels containing Brazilian-specific high-coverage whole-genome data remain unavailable. Evidence suggests that LD patterns unique to Brazilians can substantially affect imputation performance. Prior work has demonstrated that incorporating high-density array data from Brazilian individuals increases the number of successfully imputed variants and enhances imputation quality compared with using the 1000 Genomes Project panel alone (41).

PRS accuracy is sensitive to imputation quality, especially for variants with low allele frequency or weaker tagging. Therefore, insufficient marker recovery in Brazilian genomes could have attenuated the performance of European-derived PRS in our study. Importantly, our analytical design—using identical imputation pipelines and downstream PRS computation across samples—isolates the lack of a Brazilian-specific reference imputation panel as the main technical limitation, independent of genotyping array. Rather than representing a limitation specific to our dataset, this challenge reflects a real-world barrier faced when computing PRS in the Brazilian population.

### 4.5 Polygenic scores derived from Brazilian data are potentially equitable for use across the entire country

Brazil is a vast continental country, with diverse social, economic and cultural aspects. Geographic location, self-identified race and ancestry background are often considered as social determinants of health in the Brazilian context. However, in our study, the Brazilian population appeared more homogeneous than expected with respect to PRS performance. Differences between São Paulo and North Minas Gerais were minor compared to the much larger gap between Brazilian cohorts and the UKB. Self-identified race and genetic ancestry did not meaningfully stratify PRS performance in Brazilians. We did not, therefore, identify significant barriers for equitable use of polygenic scores across the Brazilian population, corroborating the possibility of deriving and validating polygenic scores on individual-level Brazilian data.

## 5 Study limitations

The British sample used for PRS performance comparisons was drawn from the UKB and may overlap with cohorts used for PRS derivation or validation. Such overlap could lead to inflated PRS effect estimates in the UKB, potentially amplifying contrasts with the Brazilian samples. Importantly, this limitation does not impact the central finding of this study, namely the limited transferability of UKB–derived PRS to Brazilian populations.

## 6 Conclusion

Readily available UKB-based PRS are not directly transferable to the Brazilian population. Portability varies across traits, and validated scores yield weaker effects. Empirical testing of European-based PRS in Brazilian cohorts is therefore necessary before clinical or research use. Regional origin, self-identified race, and genetic similarity do not consistently identify subgroups with differential score performance. There is an urgent need to incorporate genomic Brazilian data in polygenic score development cohorts.

## Data Availability

The original contributions presented in the study are included in the article/Supplementary material, further inquiries can be directed to the corresponding author.

## Supplementary material

Supplementary Figure 1 Effect size of polygenic risk scores validated in the UKB, Brazilian White and Brazilian Non-White subgroups. **(A)** Thirty validated BMI polygenic scores. **(B)** Fifteen validated SBP scores. **(C)** Twenty validated DBP scores. Dots represent the linear regression coefficients (betas). The vertical bars correspond to the 95% confidence intervals. PRS raw values were standardized to z-score units; betas reflect the change in the outcome per one standard deviation increase in the PRS. BMI, body mass index. CI, confidence interval. DPB, diastolic blood pressure. PRS, polygenic risk score. SBP, systolic blood pressure. UKB, United Kingdom Biobank.

Supplementary Figure 2 Effect size of polygenic risk scores validated in the UKB and in the Brazilian UMAP subgroups. Subgroup Brazil 1 includes Brazilians sharing genetic ancestry similar to the British, inferred by location within UMAP dimension 2 ≤ -2.5 (*n* = 404). Subgroup Brazil 2 comprises Brazilian participants genetically distant to the UK sample, defined by location beyond UMAP dimension 2 > - 2.5 (*n* = 2,768). **(A)** Thirty validated BMI polygenic scores. **(B)** Fifteen validated SBP scores. **(C)** Twenty validated DBP scores. Dots represent the linear regression coefficients (betas). The vertical bars correspond to the 95% confidence intervals. PRS raw values were standardized to z-score units; betas reflect the change in the outcome per one standard deviation increase in the PRS. BMI, body mass index. CI, confidence interval. DPB, diastolic blood pressure. PRS, polygenic risk score. SBP, systolic blood pressure. UKB, United Kingdom Biobank. UMAP, uniform manifold approximation and projection.

Supplementary Figure 3 Effect size of polygenic risk scores validated in the UKB and in the Brazilian PCA subgroups. Subgroup Brazil 1 includes Brazilians sharing genetic ancestry similar to the British, delimited by PC1 ≤ -0.01 and PC2 ≤ 0.00 (*n* = 390). Subgroup Brazil 2 comprises Brazilian participants genetically distant to the UK sample, defined by PC1 > -0.01 or PC2 > 0.00 (*n* = 2,782). **(A)** Thirty-one validated BMI polygenic scores. **(B)** Fourteen validated SBP scores. **(C)** Nineteen validated DBP scores. Dots represent the linear regression coefficients (betas). The vertical bars correspond to the 95% confidence intervals. PRS raw values were standardized to z-score units; betas reflect the change in the outcome per one standard deviation increase in the PRS. BMI, body mass index. CI, confidence interval. DPB, diastolic blood pressure. PCA, principal component analysis. PRS, polygenic risk score. SBP, systolic blood pressure. UKB, United Kingdom Biobank.

Supplementary Figure 4 Odds ratio for obesity and hypertension of BMI and blood pressure PRS in the UKB and in the Brazilian samples. PRS distributions were divided into quintiles, defining risk categories specific to each sample. Individuals in the highest (5th) risk quintile were compared to those in the lowest (1st) quintile. **(A)** Twenty-nine BMI PRS associated with obesity in all three samples. **(B)** Fifteen SBP PRS associated with hypertension. **(C)** Fifteen DBP PRS associated with hypertension. Dots represent the OR and the vertical bars correspond to the 95% confidence intervals. BMI, body mass index. CI, confidence interval. DPB, diastolic blood pressure. OR, odds ratio. PRS, polygenic risk score. SBP, systolic blood pressure. UKB, United Kingdom Biobank.
