# Supplementary material for: Limited transferability of European-based body mass index and blood pressure polygenic scores to admixed Brazilian cohorts
Source: Front Med (Lausanne). 2026 Mar 13;13:1771205. doi: 10.3389/fmed.2026.1771205 (PMC13021482; doi:10.3389/fmed.2026.1771205)
Supplement: Supplementary file 2 [file Table_2.docx]

Supplementary table 2 – Population baseline characteristics.

|  | **UK Biobank** | **São Paulo** | **North Minas Gerais** | **p value** |
| --- | --- | --- | --- | --- |
| **Male** | 707 (44.6) | 716 (45.1) | 521 (32.8) | < 0.001 |
| **Age, yr** | 59 (51 – 64) **[A]** | 51 (45 – 57) **[B]** | 58 (49 – 68) **[A]** | < 0.001 |
| **Body mass index, kg/m^2^** | 26.8 (24.1 – 29.7) **[A]** | 26.7 (24.1 – 29.7) **[A]** | 25.3 (22.2 – 28.8) **[B]** | < 0.001 |
| **Systolic blood pressure, mmHg** | 136.5 (125.5 – 151) **[A]** | 122 (113 – 133) **[B]** | 131 (118 – 149) **[C]** | < 0.001 |
| **Diastolic blood pressure, mmHg** | 82 (75.5 – 88.5) **[A]** | 74 (68 – 81) **[B]** | 77 (69 – 85) **[C]** | < 0.001 |
| **Obesity** | 374 (23.6) | 386 (24.3) | 284 (17.9) | < 0.001 |
| **Hypertension** | 376 (23.7) | 503 (31.7) | 993 (62.6) | < 0.001 |
| **Self-identified race group** |  |  |  | < 0.001 |
| White (“branco”) | 1586 (100%) | 904 (57.0) | 337 (21.2) | < 0.001 |
| Black (“negro”) | - | 201 (12.7) | 282 (17.8) | < 0.001 |
| Mixed (“pardo”) | - | 374 (23.6) | 934 (58.9) | < 0.001 |
| Indigenous (“indígena”) | - | 73 (4.6) | 4 (0.3) | < 0.001 |
| Asian (“amarelo”) | - | 17 (1.1) | 23 (1.5) | 0.008 |
| Not reported | - | 17 (1.1) | 6 (0.4) |  |

Continuous variables are presented as medians with interquartile ranges and compared using Kruskal-Wallis followed by Dunn test. Groups not sharing the same letter are statistically different. Categorical variables are described as counts and percentage and compared using Chi-square test. Comparisons of self-identified race include only the Brazilian samples. UK, United Kingdom.
