# Supplementary material for: Limited transferability of European-based body mass index and blood pressure polygenic scores to admixed Brazilian cohorts
Source: Front Med (Lausanne). 2026 Mar 13;13:1771205. doi: 10.3389/fmed.2026.1771205 (PMC13021482; doi:10.3389/fmed.2026.1771205)
Supplement: Supplementary file 3 [file Table_3.docx]

Supplementary Table 3 - PRS diagnostic performance for obesity and hypertension, with sensitivity, specificity, negative predictive value, positive predictive value, and AUPRC.

| **Obesity, BMI PRS (PGS002842)** | | | | | |
| --- | --- | --- | --- | --- | --- |
| **Sample** | **Sensitivity** | **Specificity** | **PPV** | **NPV** | **AUPRC** |
| UK Biobank | 0.602 | 0.769 | 0.457 | 0.860 | 0.525 |
| São Paulo | 0.765 | 0.377 | 0.284 | 0.843 | 0.325 |
| North Minas Gerais | 0.602 | 0.479 | 0.231 | 0.834 | 0.261 |
| **Hypertension, SBP PRS (PGS002807)** | | | | | |
| **Sample** | **Sensitivity** | **Specificity** | **PPV** | **NPV** | **AUPRC** |
| UK Biobank | 0.708 | 0.495 | 0.307 | 0.852 | 0.373 |
| São Paulo | 0.848 | 0.252 | 0.346 | 0.780 | 0.373 |
| North Minas Gerais | 0.983 | 0.0287 | 0.632 | 0.403 | 0.679 |
| **Hypertension, DBP PRS (PGS002639)** | | | | | |
| **Sample** | **Sensitivity** | **Specificity** | **PPV** | **NPV** | **AUPRC** |
| UK Biobank | 0.776 | 0.323 | 0.268 | 0.825 | 0.323 |
| São Paulo | 0.790 | 0.321 | 0.356 | 0.775 | 0.407 |
| North Minas Gerais | 0.974 | 0.0389 | 0.629 | 0.477 | 0.683 |

PRS association with obesity and hypertension was tested in a univariate logistic regression model. Samples were randomly split into training and test sets. Results are the mean of the metrics obtained from test sets in one hundred iterations. BMI, body mass index. DBP, diastolic blood pressure. AUPRC, area under the precision-recall curve. NVP, negative predictive value. PPV, positive predictive value. PRS, polygenic risk score. SBP, systolic blood pressure.
