# Supplementary figures and images for: Limited transferability of European-based body mass index and blood pressure polygenic scores to admixed Brazilian cohorts
Source: Front Med (Lausanne). 2026 Mar 13;13:1771205. doi: 10.3389/fmed.2026.1771205 (PMC13021482; doi:10.3389/fmed.2026.1771205)

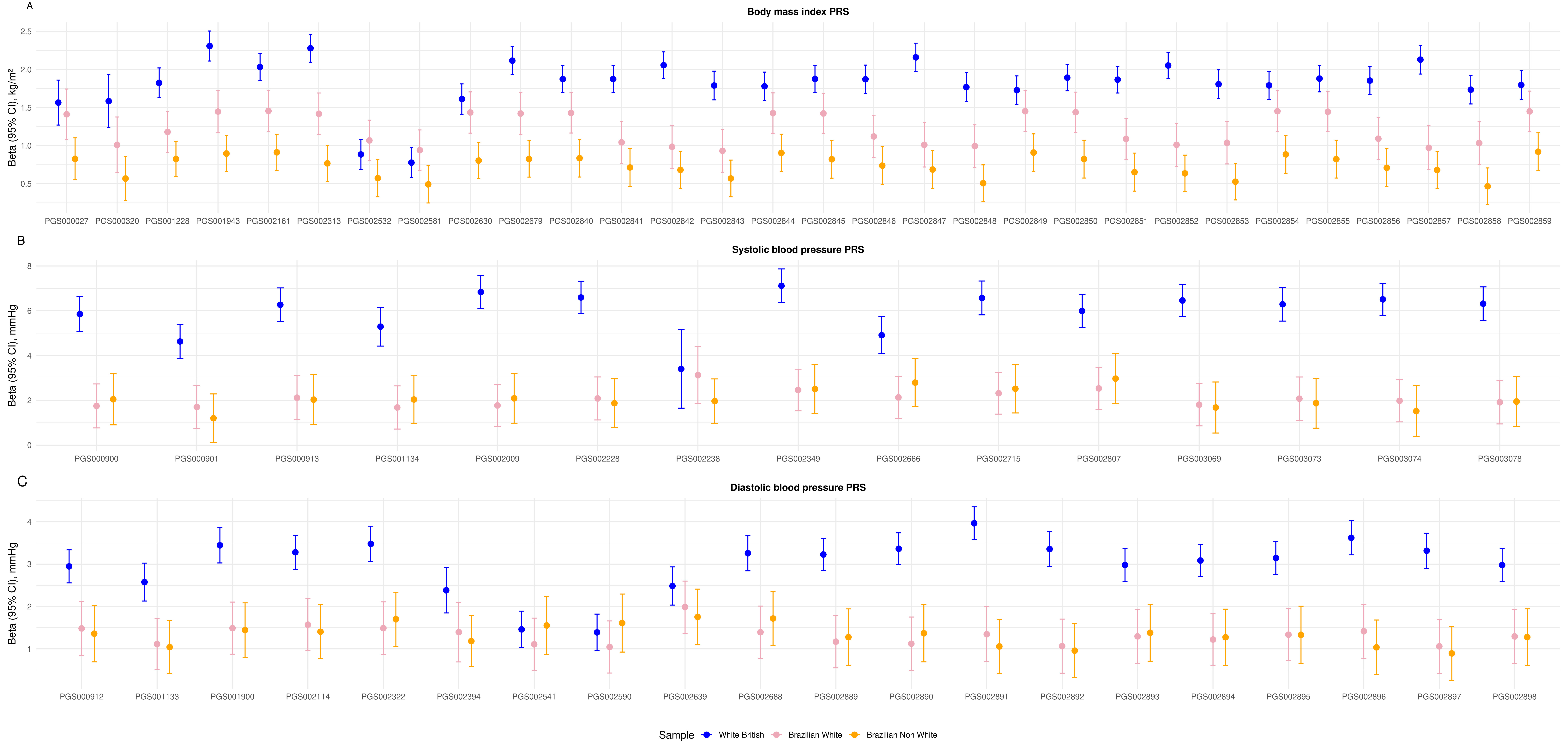

Supplement: Supplementary Figure 1 — Effect size of polygenic risk scores validated in the UKB, Brazilian White and Brazilian Non-White subgroups. (A) Thirty validated BMI polygenic scores. (B) Fifteen validated SBP scores. (C) Twenty validated DBP scores. Dots represent the linear regression coefficients (betas). The vertical bars correspond to the 95% confidence intervals. PRS raw values were standardized to z-score units; betas reflect the change in the outcome per one standard deviation increase in the PRS. BMI, body mass index. CI, confidence interval. DPB, diastolic blood pressure. PRS, polygenic risk score. SBP, systolic blood pressure. UKB, United Kingdom Biobank. [file Image_1.jpeg]

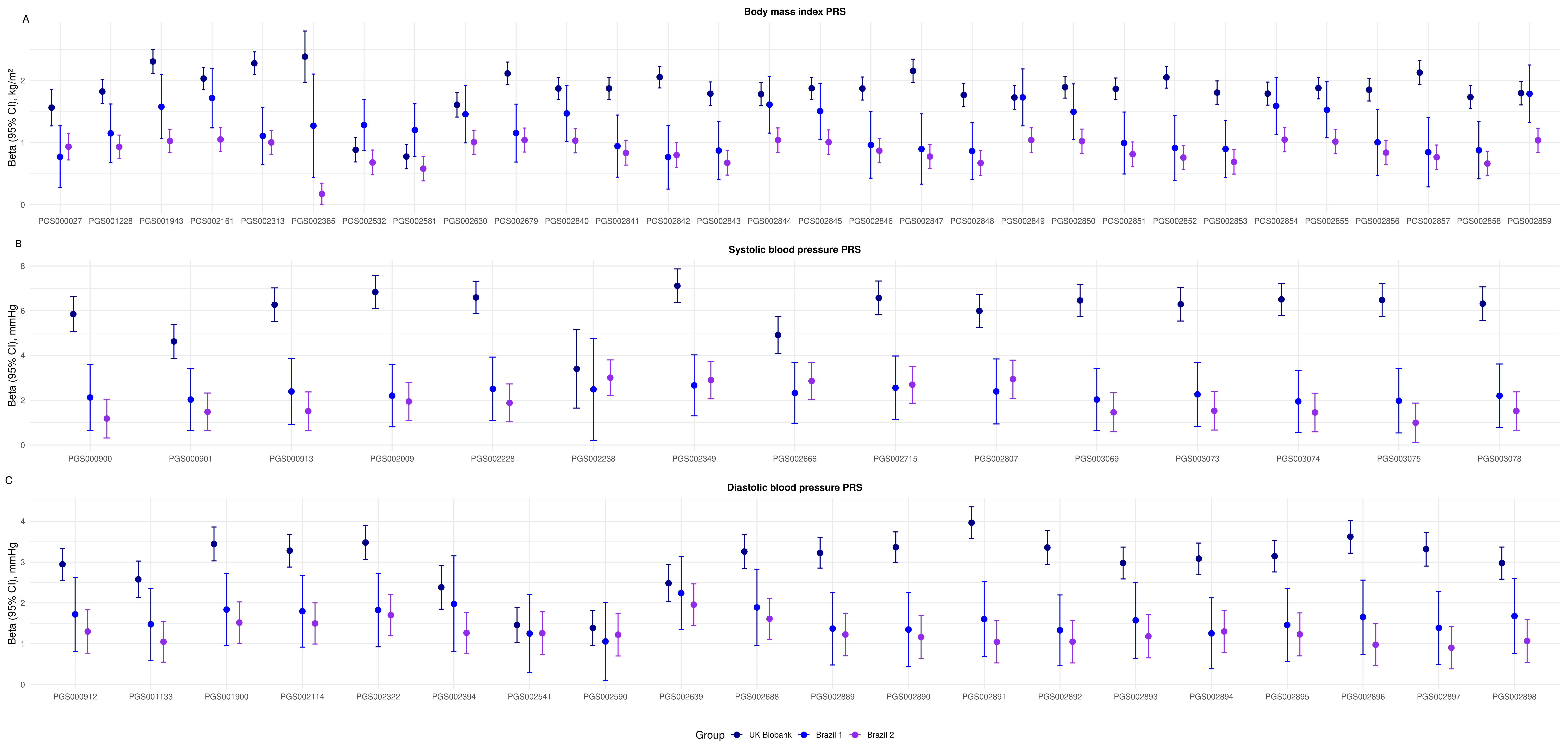

Supplement: Supplementary Figure 2 — Effect size of polygenic risk scores validated in the UKB and in the Brazilian UMAP subgroups. Subgroup Brazil 1 includes Brazilians sharing genetic ancestry similar to the British, inferred by location within UMAP dimension 2 ≤ -2.5 (n = 404). Subgroup Brazil 2 comprises Brazilian participants genetically distant to the UK sample, defined by location beyond UMAP dimension 2 > - 2.5 (n = 2,768). (A) Thirty validated BMI polygenic scores. (B) Fifteen validated SBP scores. (C) Twenty validated DBP scores. Dots represent the linear regression coefficients (betas). The vertical bars correspond to the 95% confidence intervals. PRS raw values were standardized to z-score units; betas reflect the change in the outcome per one standard deviation increase in the PRS. BMI, body mass index. CI, confidence interval. DPB, diastolic blood pressure. PRS, polygenic risk score. SBP, systolic blood pressure. UKB, United Kingdom Biobank. UMAP, uniform manifold approximation and projection. [file Image_2.jpeg]

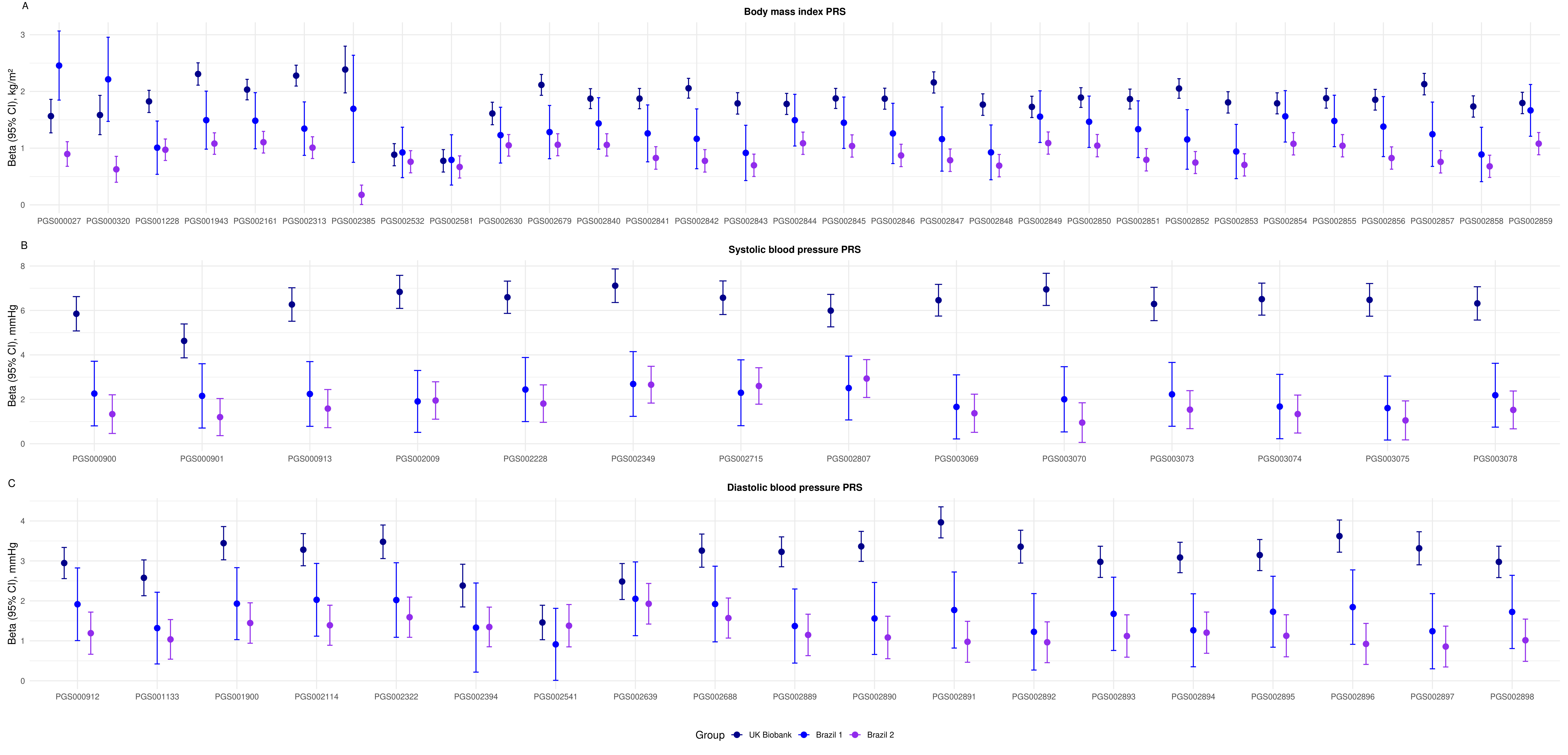

Supplement: Supplementary Figure 3 — Effect size of polygenic risk scores validated in the UKB and in the Brazilian PCA subgroups. Subgroup Brazil 1 includes Brazilians sharing genetic ancestry similar to the British, delimited by PC1 ≤ -0.01 and PC2 ≤ 0.00 (n = 390). Subgroup Brazil 2 comprises Brazilian participants genetically distant to the UK sample, defined by PC1 > -0.01 or PC2 > 0.00 (n = 2,782). (A) Thirty-one validated BMI polygenic scores. (B) Fourteen validated SBP scores. (C) Nineteen validated DBP scores. Dots represent the linear regression coefficients (betas). The vertical bars correspond to the 95% confidence intervals. PRS raw values were standardized to z-score units; betas reflect the change in the outcome per one standard deviation increase in the PRS. BMI, body mass index. CI, confidence interval. DPB, diastolic blood pressure. PCA, principal component analysis. PRS, polygenic risk score. SBP, systolic blood pressure. UKB, United Kingdom Biobank. [file Image_3.jpeg]

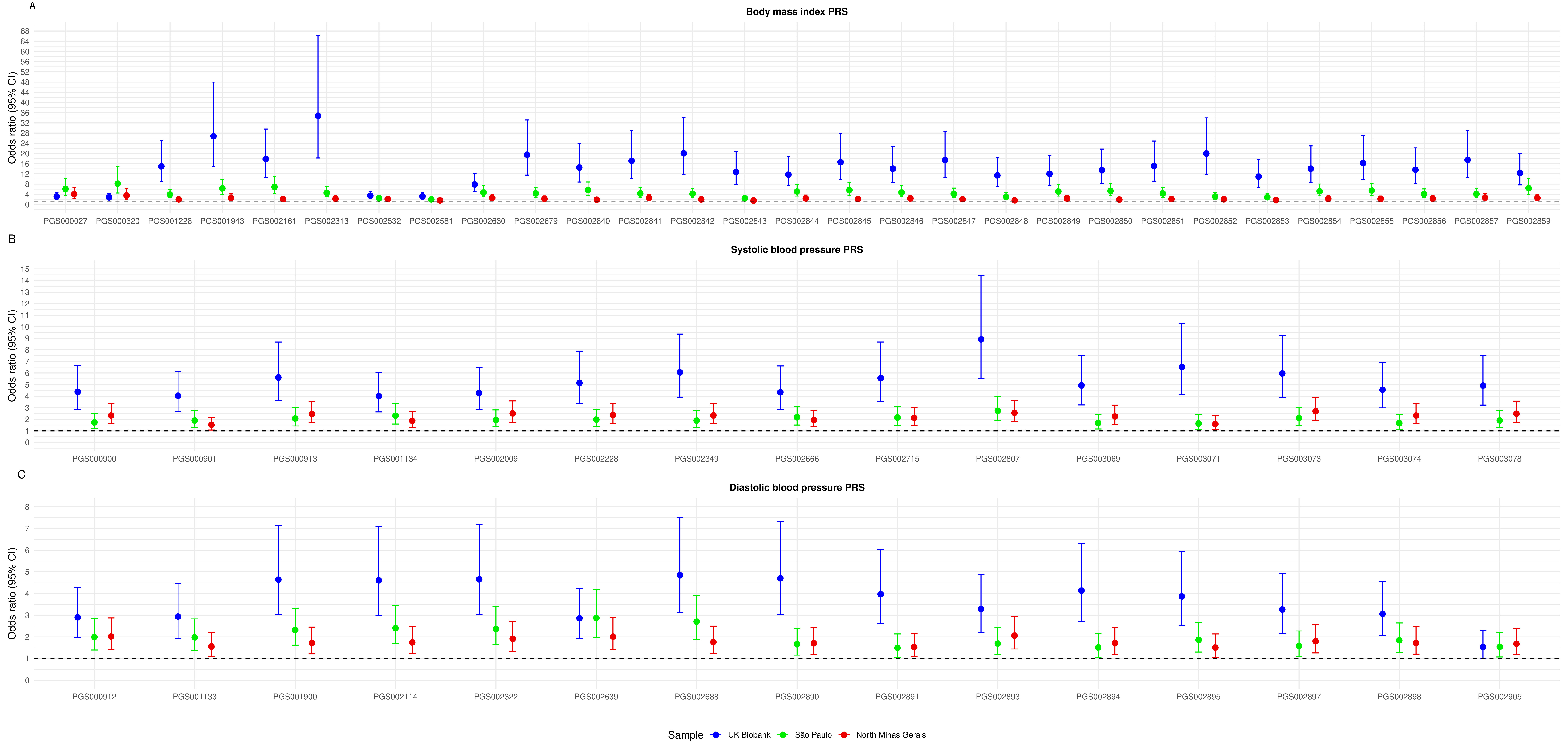

Supplement: Supplementary Figure 4 — Odds ratio for obesity and hypertension of BMI and blood pressure PRS in the UKB and in the Brazilian samples. PRS distributions were divided into quintiles, defining risk categories specific to each sample. Individuals in the highest (5th) risk quintile were compared to those in the lowest (1st) quintile. (A) Twenty-nine BMI PRS associated with obesity in all three samples. (B) Fifteen SBP PRS associated with hypertension. (C) Fifteen DBP PRS associated with hypertension. Dots represent the OR and the vertical bars correspond to the 95% confidence intervals. BMI, body mass index. CI, confidence interval. DPB, diastolic blood pressure. OR, odds ratio. PRS, polygenic risk score. SBP, systolic blood pressure. UKB, United Kingdom Biobank. [file Image_4.jpeg]
